# Assessing the Effects of *Aedes aegypti kdr* Mutations on Pyrethroid Resistance and Its Fitness Cost

**DOI:** 10.1371/journal.pone.0060878

**Published:** 2013-04-08

**Authors:** Luiz Paulo Brito, Jutta G. B. Linss, Tamara N. Lima-Camara, Thiago A. Belinato, Alexandre A. Peixoto, José Bento P. Lima, Denise Valle, Ademir J. Martins

**Affiliations:** 1 Laboratório de Fisiologia e Controle de Artrópodes Vetores, Instituto Oswaldo Cruz, FIOCRUZ, Rio de Janeiro, RJ, Brasil; 2 Laboratório de Biologia Molecular de Insetos, Instituto Oswaldo Cruz, FIOCRUZ, Rio de Janeiro, RJ, Brasil; 3 Instituto Nacional de Ciência e Tecnologia em Entomologia Molecular, Rio de Janeiro, RJ, Brasil; Tulane University, United States of America

## Abstract

Pyrethroids are the most used insecticide class worldwide. They target the voltage gated sodium channel (Na_V_), inducing the knockdown effect. In *Aedes aegypti*, the main dengue vector, the AaNa_V_ substitutions Val1016Ile and Phe1534Cys are the most important knockdown resistance (*kdr*) mutations. We evaluated the fitness cost of these *kdr* mutations related to distinct aspects of development and reproduction, in the absence of any other major resistance mechanism. To accomplish this, we initially set up 68 crosses with mosquitoes from a natural population. Allele-specific PCR revealed that one couple, the one originating the CIT-32 strain, had both parents homozygous for both *kdr* mutations. However, this pyrethroid resistant strain also presented high levels of detoxifying enzymes, which synergistically account for resistance, as revealed by biological and biochemical assays. Therefore, we carried out backcrosses between CIT-32 and Rockefeller (an insecticide susceptible strain) for eight generations in order to bring the *kdr* mutation into a susceptible genetic background. This new strain, named Rock-kdr, was highly resistant to pyrethroid and presented reduced alteration of detoxifying activity. Fitness of the Rock-kdr was then evaluated in comparison with Rockefeller. In this strain, larval development took longer, adults had an increased locomotor activity, fewer females laid eggs, and produced a lower number of eggs. Under an inter-strain competition scenario, the Rock-kdr larvae developed even slower. Moreover, when Rockefeller and Rock-kdr were reared together in population cage experiments during 15 generations in absence of insecticide, the mutant allele decreased in frequency. These results strongly suggest that the *Ae. aegypti kdr* mutations have a high fitness cost. Therefore, enhanced surveillance for resistance should be priority in localities where the *kdr* mutation is found before new adaptive alleles can be selected for diminishing the *kdr* deleterious effects.

## Introduction

Diseases like dengue, malaria, lymphatic filariasis, leishmaniasis and Chagas’ disease are caused by pathogens transmitted by insect vectors and represent a significant part of all morbidity and mortality records in tropical countries. In the last decades, urban areas in most of these countries have faced an accelerated and disorganized growth, with deficient sanitation and general infrastructure, a scenario that favors the expansion of insect vector populations [1]. Since there are still no effective vaccines against these etiologic agents, disease control strongly relies on actions against their insect vectors. In this sense, insecticides are expected to remain a key component in the control of insect populations for a long time [2].

*Aedes aegypti* is the main dengue vector, currently the most important arthropod-borne viral infection of man [3]. Brazil is hyperendemic for the dengue virus, with confirmed co-circulation of the four serotypes since 2010 when more than one million cases of the disease were registered [4]. In this country, *Ae. aegypti* control is increasingly based on community participation campaigns that strengthen the importance of mechanical control through the elimination of potential breading sites. Thousands of health agents are responsible for visiting residences and orienting dwellers. Ideally, treatment with larvicides takes place, in 4–6 annual cycles as a complementary measure, only on water reservoirs that cannot be eliminated. Presently two different larvicides are employed, temephos or, in localities with confirmed resistance to this organophosphate, a chitin synthesis inhibitor. Control of adult mosquitoes, performed by ultra-low volume (ULV) space spraying of pyrethroids, is theoretically restricted to epidemic seasons. However, the domestic use of this insecticide class is massive, both through aerosol cans or even space spraying services hired privately by residential complexes. At a global scale, pyrethroids are by far the most popular insecticide class, in terms of surface area [5], against adult mosquitoes since they act very rapidly (the knockdown effect), are easily applied and less harmful to both the environment and man. Up to now, they are also the only class of products recommended by the World Health Organization (WHO) for use in insecticide treated materials (ITMs), which are currently largely distributed for personal protection against malaria and dengue vectors [6], [7]. However, insecticide resistant populations are spreading all over the world, representing a growing obstacle to vector control programs [8].

*Aedes aegypti* pyrethroid resistance may be the consequence of the selection of detoxifying enzymes with altered expression, mainly from the multi function oxidases (MFO) [9] and glutathione-S transferases (GST) super-families [10], but also esterases (EST) [11]. Nevertheless, point mutations in the molecular pyrethroid target site in the mosquito central nervous system, the voltage gated sodium channel (Na_V_), generally referred to as *kdr* mutations, are the major reported causes of resistance to this insecticide class [12]. Na_V_ is a transmembrane protein present in the neuronal axons and composed of four homologous domains (I-IV) each with six hydrophobic segments (S1–S6) [13]. In many insect species, mutations related to both pyrethroid and DDT resistance are placed mainly in the IIS6 region. The replacement of a Leu for a Phe in the 1014 site (Leu1014Phe), first described in a DDT resistant *Musca domestica* strain [14], is the most common. Besides, other substitutions in the same homologous site have been described in a series of species, such as Leu1014Ser in the mosquitoes *Anopheles gambiae* [15] and *Culex pipiens* [16] and Leu1014His in the tobacco budworm *Heliothis virescens* [17]. In *Ae. aegypti*, these substitutions at the 1014 site are unlikely to occur since two independent changes in the same codon would be necessary [18]. Instead, mutations in different positions have been observed in *Ae. aegypti* populations from Latin America and Southeast Asia. At least two sites are indeed related to pyrethroid resistance, 1016 (Val to Ile or Gly) and 1534 (Phe to Cys) in the IIS6 and IIIS6 segments, respectively [18]–[22]. There is also another polymorphic site in the IIS6 region, 1011 (Ile to Met or Val), but its relative role in pyrethroid resistance remains to be elucidated [19], [21]. Recent reports from Brazil and Mexico reveal a fast dissemination of resistance to pyrethroids together with a drastic increase in the rates of the Val1016Ile mutation in *Ae. aegypti* populations [7], [20], [23]. Selection pressure under laboratory conditions confirmed this tendency [18], [24].

In an evolutionary perspective, selection for insecticide resistance may lead to a series of side effects in the life history traits of an insect population. This may be achieved as a result of pleiotropic effects in the resistance genes themselves or as a consequence of a hitchhiking effect, where certain alleles of non-related loci increase in frequency in consequence of a strong linkage to a resistance gene under directional selection [25]. In some cases these effects may be associated with a reduced fitness in the absence of insecticide [26]. Consequently, there is a decrease in the vector population resistance levels after the insecticide is withdrawn. Ultimately, this could contribute to a rational utilization of a given insecticide, here represented by the possibility of its re-introduction once susceptible levels are recovered by the vector population. In this sense, knowledge of the resistance status and its underlying mechanisms are the first steps toward a more effective resistance management. In addition, the fitness cost of insecticide resistance should be more thoroughly explored in a comparative perspective, both in the presence and in the absence of the insecticide. Herein, we present a study of the effect of the *Aedes aegypti kdr* mutations on several life-history parameters, in order to evaluate the fitness cost. We also examine the changes in mutant allele frequencies along 15 generations in population cages without insecticide selection pressure.

## Results

### 1. Establishment and Characterization of a kdr Homozygous Strain

**Figure 1:**
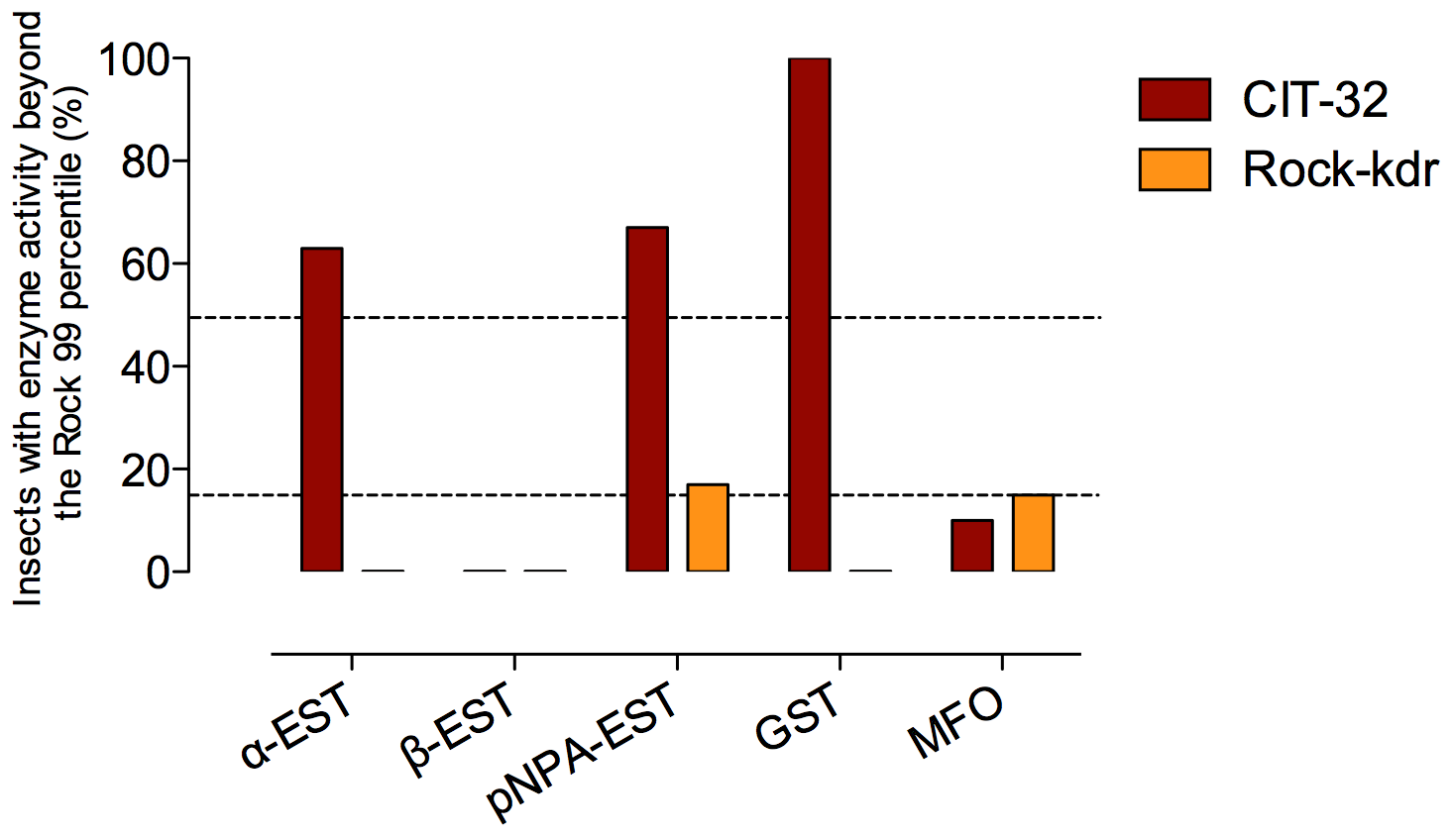
Activity of enzymes related to insecticide metabolic resistance in *Aedes aegypti* strains. The cut-offs are (dashed lines) determined by the Rockefeller 99 percentile value of each enzyme (see [11]). Rockefeller is a reference strain of insecticide susceptibility and vigor. Distributions with less than 15% of individuals beyond the cut-off are considered unaltered. Between 15 and 50% are altered and above 50% are highly altered. CIT-32 is the original *kdr* strain, derived from a pyrethroid resistant Brazilian *Aedes aegypti* population. Rock-kdr is the *kdr* strain, backcrossed for eight generations with Rockefeller in order to reduce the contribution of detoxification enzymes to pyrethroid resistance.

We initially assembled 68 crosses recruiting insects from a natural population of *Ae. aegypti* from Cachoeiro do Itapemirim, a Brazilian municipality. Allele specific PCR (see Methods) revealed that both parents of strain CIT-32 had genotype 1011 Ile/Ile (wild) +1016 Ile/Ile (mutant) for the AaNa_V_. However, besides homozygous for alteration in the pyrethroid target site, this strain displayed highly altered activity of enzymes related to metabolic resistance, particularly α-EST, pNPA-EST and GST, when compared to the susceptible reference Rockefeller strain, Rock (Figure 1, CIT-32). Since we aimed to determine the actual role of the *kdr* mutation on resistance and assess its fitness cost, it was necessary to reduce the contribution of metabolic resistance. Therefore, we carried out backcrosses between CIT-32 and Rockefeller (Rock), a laboratory reference strain, for eight generations in order to bring the *kdr* mutation into the Rock susceptible genetic background, identifying the heterozygotes from each cross by allele specific PCR (see Methods). Afterwards, 1016 Ile/Ile homozygous individuals were produced by crosses between heterozygous parents, originating the Rock-kdr strain. This procedure resulted in a considerable decrease of the insecticide metabolic resistance of Rock-kdr as compared to CIT-32 (Figure 1). One exception was observed for MFO, an enzyme class exhibiting a slightly higher rate of altered individuals in Rock-kdr than in CIT-32.

It is of note that in the course of this study after establishing the CIT-32 and Rock-kdr strains we further evaluated the Na_V_ 1534 site, carrying an additional substitution, Phe/Cys, recently related to pyrethroid resistance in *Ae. aegypti* natural populations from Grand Cayman and Thailand [22], [27]. We confirmed that both strains were also homozygous for the mutation in that site indicating that they were probably in linkage disequilibrium in the original population.

#### Bioassay

**Figure 2:**
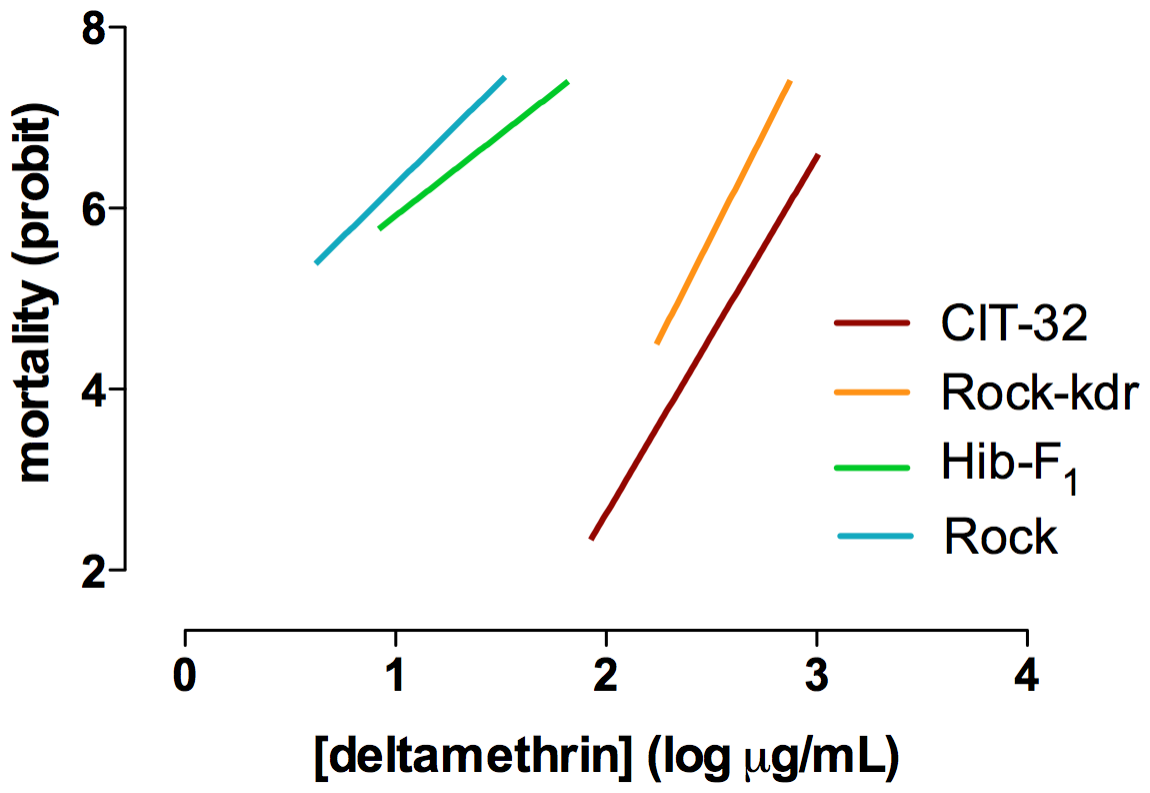
Linear regression curves of *Aedes aegypti* mortality after exposure to deltamethrin impregnated papers. Strains evaluated correspond to the susceptibility control (Rock), the 1016 Ile/Ile selected strains with genetic background from a natural population (CIT-32) or from Rock (Rock-kdr), and the F1 offspring between Rock-kdr and Rock (Hib-F_1_).

Dose-response bioassays with deltamethrin-impregnated papers showed that both Rock and the heterozygous Hib-F1 mosquitoes, derived from CIT-32 and Rock crosses, share a susceptible profile while CIT-32 and Rock-kdr strains exhibit high resistant levels (Figure 2). This result corroborates the recessive nature of the *kdr* mutations for pyrethroid resistance. Besides, since in Rock-kdr the insecticide metabolic resistance has been greatly weakened, the similarity of Rock-kdr and CIT-32 pyrethroid resistant profiles indicates that most of the resistance can be attributed to the *kdr* allele.

### 2. Fitness Cost – Developmental Parameters

#### Larval development time and pupae formation rate

**Figure 3:**
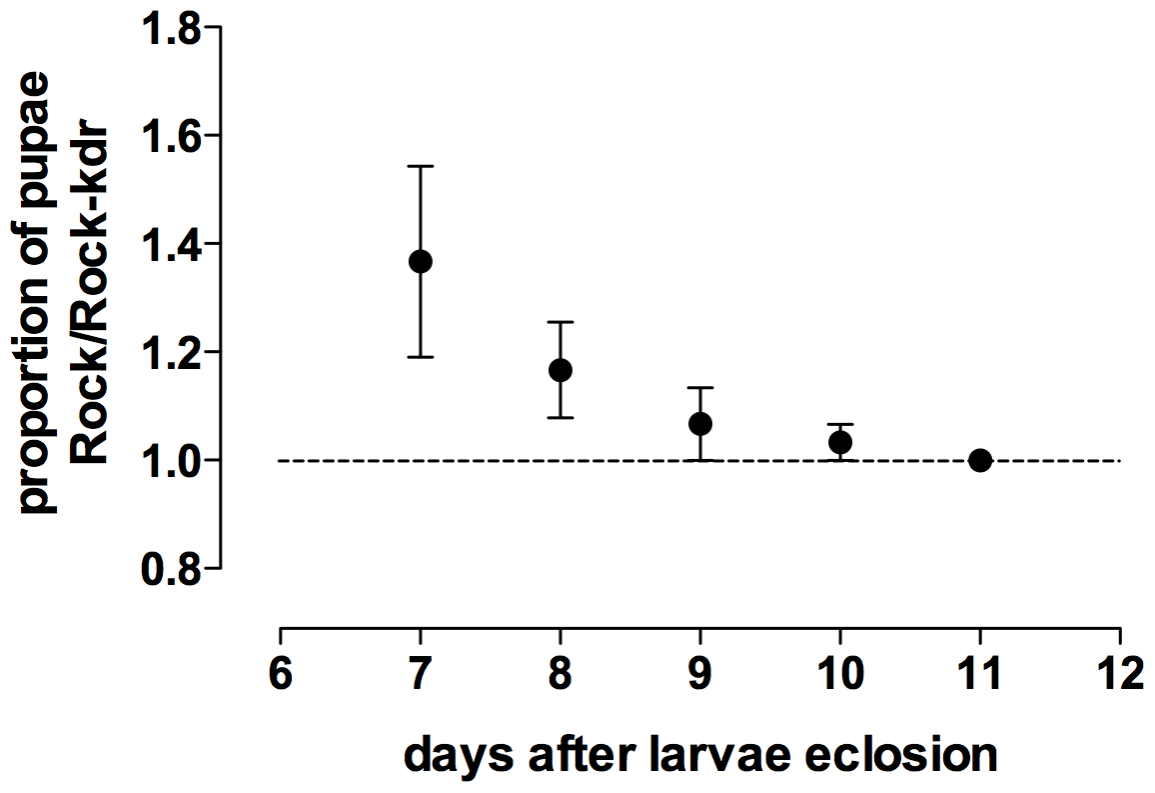
Comparison of larval development time between Rockefeller and Rock-kdr *Ae. aegypti* strains. Numbers represent the cumulative daily proportion of Rock and Rock-kdr pupae formation after larvae eclosion under standard laboratory conditions. SEM is indicated. Gray dotted line indicates equal proportion (rate = 1) between strains.

When Rock-kdr and Rock larvae were reared under controlled conditions, the latter developed faster. Regarding pupae which managed to develop up to the seventh day after larvae eclosion, there were around 40% more Rock than Rock-kdr (Figure 3). Although resistant larvae took more time to develop, there was no significant difference in the amount of pupation, i.e. viability from egg to pupae, between strains (t = 0.3055; df = 16, p = 0.764), reached 93.9% (±1.3) and 94.6% (±2.1) for Rock-kdr and Rock, respectively.

#### Adult longevity

This parameter was evaluated under two distinct food regimens, sugar offered *ad libitum* (to males and females) or supplemented with blood feeding (females). The results are shown in Figure S1. As expected, male longevity (Figure S1-A) was lower than female (Figure S1-B). The analysis of the survival curves indicated no significant difference between Rock and Rock-kdr for both males (χ^2^ = 0.4153, df = 1, p = 0.5195) and females fed only with sugar (χ^2^ = 3.809, df = 1, p = 0.0510). Mortality of blood-fed females, for both Rock and Rock-kdr, was far lower than the females fed only with sugar. Mortality of blood-fed females reached only 12.0% (±2.8) and 6.5% (±0.7) for Rock and Rock-kdr, respectively, after 60 days of accompaniment (Figure S1-C). Their survival curves difference was also non-significant (χ^2^ = 0.8671, df = 1, p = 0.3518). Therefore, we found no evidences that the *kdr* mutations interfere with adults’ longevity.

#### Locomotor activity and circadian rhythm

**Figure 4:**
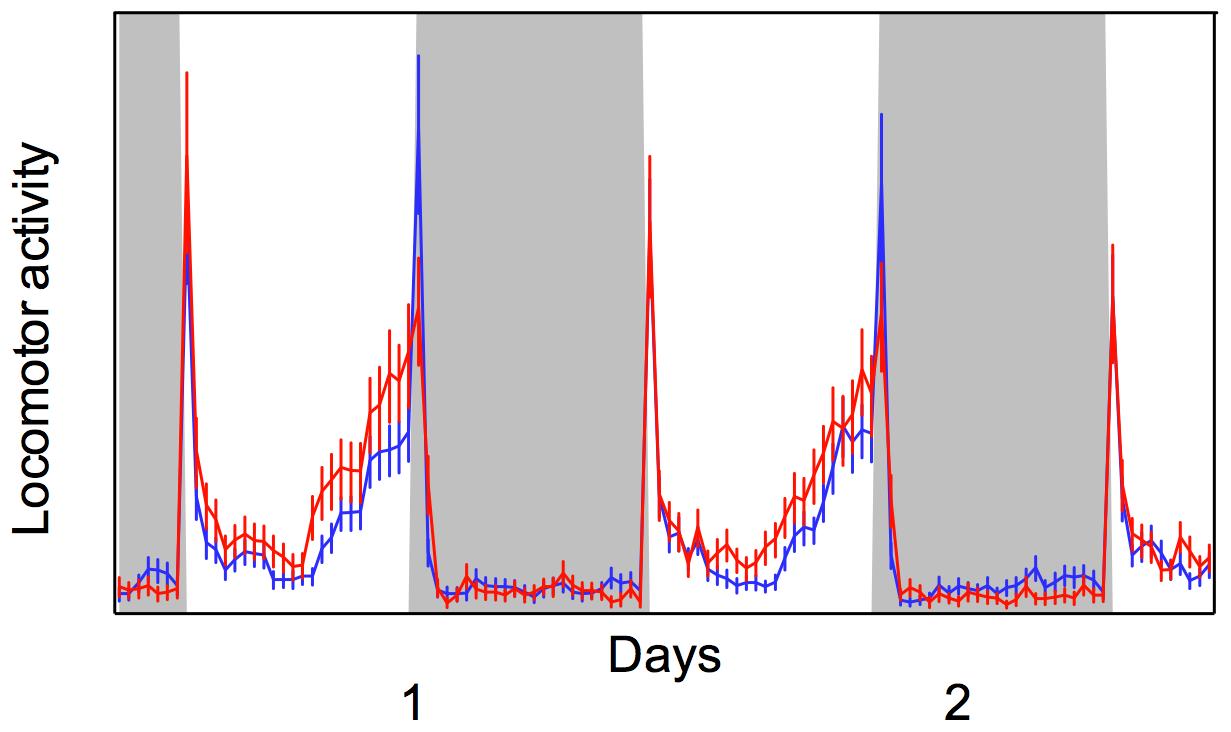
Locomotor activity Rock and Rock-kdr *Ae. aegypti* strains. Locomotor activity of susceptible (Rockefeller strain – blue line) and pyrethroid resistant (Rock-kdr – red line) *Aedes aegypti* females exposed two days under LD 12∶12, at 25°C. Dotted lines represent standard errors.

Both Rock-kdr and Rock females presented diurnal habits, with peaks of activity at the beginning and end of the photophase. Although the pattern of activity was not altered in the pyrethroid resistant strain, the level of diurnal activity (during photophase) was significantly increased (t = −2.059; df = 206; p = 0.041) in pyrethroid resistant females (Figure 4). The same result was verified when the activity during the first 30 minutes after lights-on was not considered (t = −2.11; df = 170.87; p = 0.036), meaning that this significant increase observed in the pyrethroid resistant strain during the photophase was not due to the lights-on startle response.

#### Blood feeding

The difference in average weight between Rock and Rock-kdr females before blood feeding was not significant (t = 0.3796, df = 4, p = 0.7235), indicating that there should be no differences in their total body size. Rock and Rock-kdr females respectively, engorged approximately 100 and 87% of their weight (Figure S2), this difference neither being significant (t = 0.4418, df = 4, p = 0.6815).

### 3. Fitness Cost – Reproductive Parameters

#### Female fecundity

**Figure 5:**
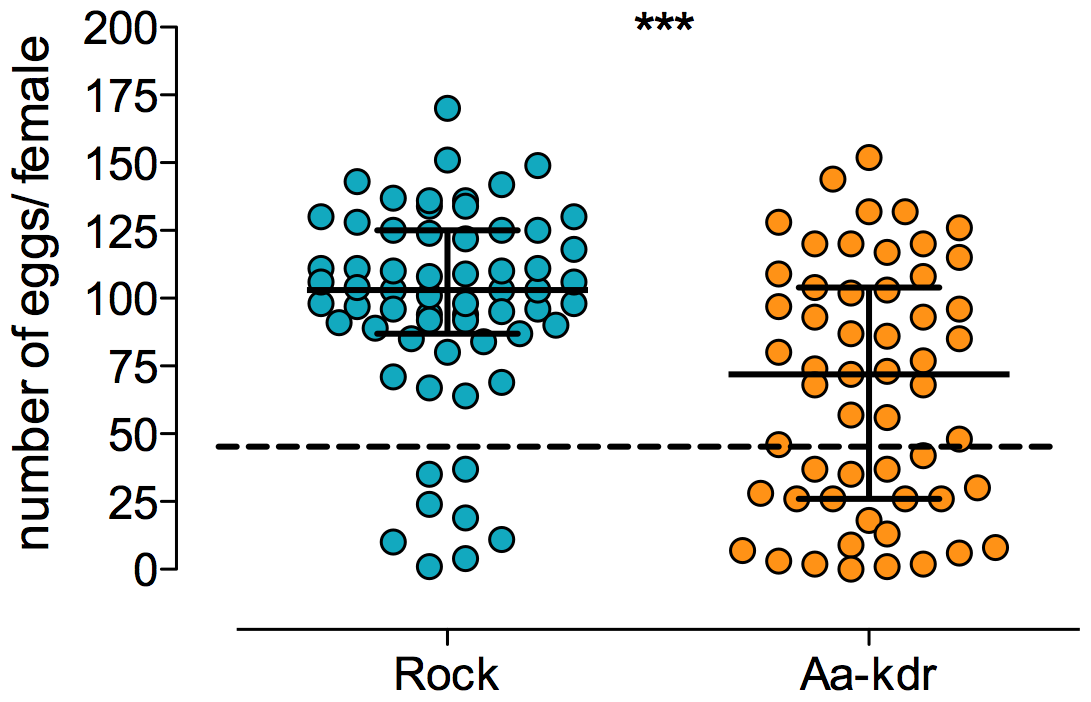
Number of eggs laid by females from Rock and Rock-kdr *Ae. aegypti* strains. Each dot represents a single female. Only females that laid at least one egg were included. Median value with interquartile range is shown for each distribution. Dotted line points 50 eggs/female, which was herein empirically considered as discriminative of successful insemination. ***Difference between strains was highly significant by *t* test.

In general, compared to Rock, fewer Rock-kdr females laid eggs and in smaller amounts (Table 1), females with few eggs or without eggs at all considered as non-inseminated [28]. In this sense, fewer Rock-kdr females must have been inseminated (χ^2^ = 13.83, df = 1, p<0.0002). Rock laid more eggs than Rock-kdr both considering exclusively females with more than 50 eggs (t = 2.580, df = 45, p<0.05) or including those with less than 50 eggs (t = 4.095, df = 74, p<0.001). The distribution of females of both Rock and Rock-kdr laying at least one egg is presented in Figure 5.

**Table 1:** Oviposition performance of Rockefeller and Rock-kdr *Ae. aegypti* females.

Females with	Rock (n)	Rock-kdr (n)
No eggs	11.1% (7)	18.8% (9)
1- 50 eggs	9.5% (6)	27.1% (13)
>50 eggs	79.4% (50)	54.2% (26)
Total (n)	63	48

#### Egg viability

The average rates (± standard deviation) of hatched larvae were 90.8 (±5.04) and 86.9 (±3.80)% for Rock and Rock-kdr, respectively. This difference was not significant (t = 1.051, df = 4, p = 0.3527).

### 4. Competition Analysis

#### Development time until adult

**Figure 6:**
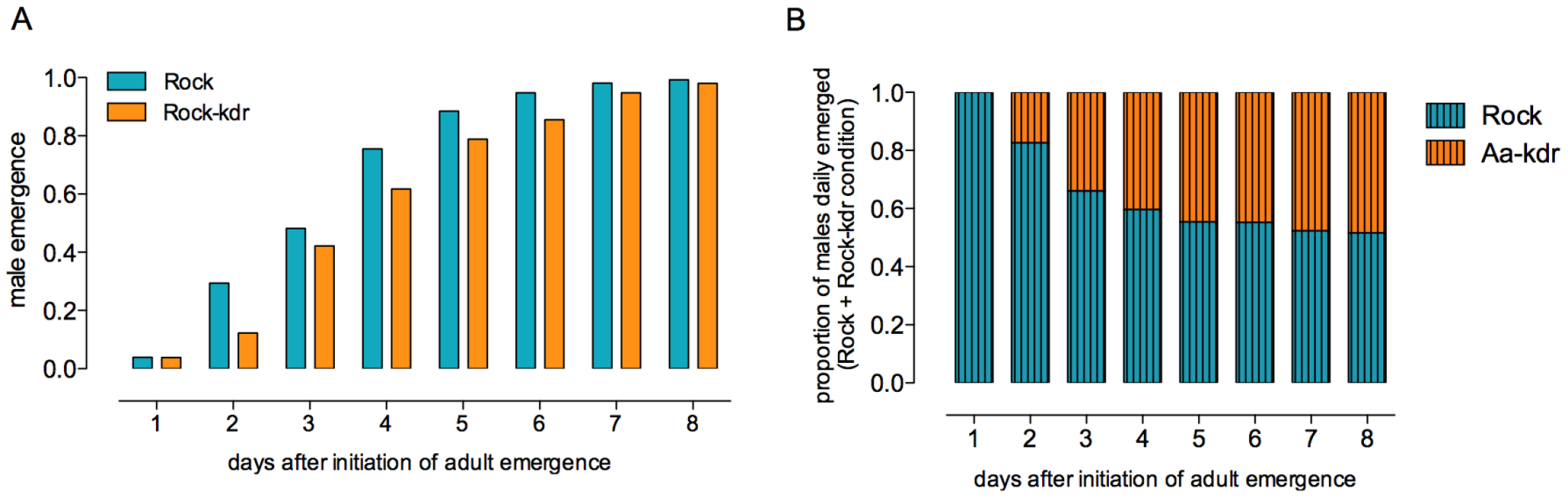
Developmental timing of *Ae. aegypti* Rock and Rock-kdr male adult emergence competing under a stringent condition. A – Cumulative rate of male emergence up to the 8th day after the beginning of adult emergence when the controls Rock and Rock-kdr were reared separately (‘intra-strain’ conditions). B – Cumulative proportion of Rock or Rock-kdr male emergence from the inter-strain competition. Male strain was daily determined by randomly genotyping 30% of emerging individuals.

As observed under standard laboratory conditions (see Figure 3), Rock-kdr took more time to develop than Rock, also with higher larval density and lower food supply (Figure 6-A). When Rock and Rock-kdr were reared together, i.e. under inter-strain competition, most of the newly emerged adult males by the fourth day were Rock (Figure 6-B). Daily numbers of emerging adults, as well as data for determination of their strain in the inter-strain condition, are presented in Tables S1 and S2.

#### Competition for insemination

A potential difference in insemination success between Rock and Rock-kdr males was tested in a pair of cages, both containing a similar number of males from each strain but only females Rock (in cage A) or Rock-kdr (in cage B). Considering that *Ae. aegypti* females mate only once, becoming refractory to further inseminations [29], if the offspring of a female was genotyped as homozygous (see Methods) it would mean that a male of the same strain was the father. In contrast, heterozygous offspring indicated insemination by a male from the other strain. For each cage, the offspring of 11 females was genotyped, and the number of homo or heterozygous offspring did not differ neither in cage A (χ^2^ = 0.1640, df = 1, p = 0.6855) nor in cage B (χ^2^ = 0.0196, df = 1, p = 0.8887). This indicates that there was no apparent insemination advantage or female preference for Rock males over Rock-kdr males.

#### Population cage experiments

**Figure 7:**
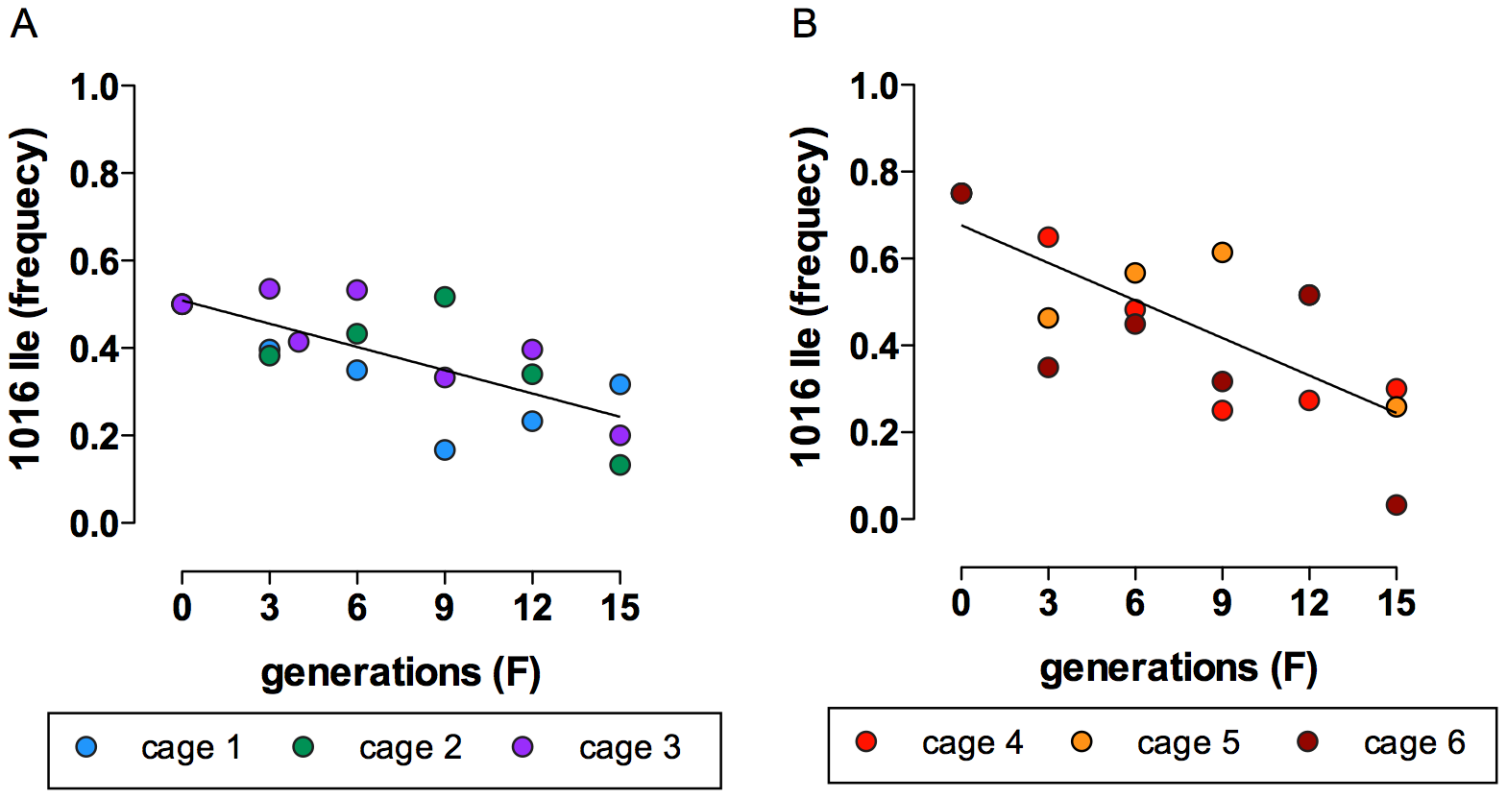
Population cage assays with pyrethroid resistant *Aedes aegypti* (Rock-kdr) and Rockefeller strains. The frequency of *AaNa_V_* alleles in the 1016 site was followed in independent cages kept under the same conditions, without insecticide exposure for 15 generations. The initial frequency of the 1016Ile *kdr* allele in cages 1–3 (A) was 0.50 and in cages 3–6 (B) was 0.75. Lines represent the linear regression analysis taken by the means of the mutant allele frequencies of the respective three cages in A (r^2^ = 0.5273, p = 0,0006) and B (r^2^ = 0,5690, p = 0,0003).

The fitness cost of the *kdr* mutations was also investigated in population cages examining the changes in the frequency of the 1016 Ile mutation, which is in complete linkage disequilibrium with the 1534 Cys mutation, over 15 non-overlapping generations under an insecticide free environment. This was performed for two initial 1016 Ile mutant allele frequencies, 50 and 75%. If the disadvantages herein noted for the Rock-kdr resulted in a real impact on fitness, one should expect that the wild allele would increase in frequency, which was indeed confirmed. In all cages, the mutant allele frequency tended to decay over the course of the 15 evaluated generations (Figure 7). When the initial 1016 Ile frequency was 50%, in the last generation the mutant allele decreased to an average frequency of 21.7% (32, 13 and 20%, respectively in cages 1, 2 and 3) (Figure 7-A). The same trend occurred in the cages where the initial frequency of the mutant allele was 75%, dropping to 30, 26 and 3%, respectively in cages 4, 5 and 6 (Figure 7-B). Detailed numbers for each cage throughout generations are presented in Table S3.

## Discussion

The diagnostic and quantification of the *kdr* mutations in *Ae. aegypti* natural populations is nowadays an important tool to predict resistance to pyrethroids in the field [18], [20], [22], [23], [30]. In many localities the mutant allele Val1016Ile was found in high frequencies with tendency of rapid increase towards fixation [20], [23]. If the mutation harbors a fitness cost in an insecticide-free environment, it is expected that once pyrethroid pressure ceases, the frequency of the mutant allele, and consequently pyrethroid resistance, will decrease. To date, evidences of physiological commitment in *Ae. aegypti kdr* mosquitoes were mainly observed in laboratory selected strains for pyrethroid resistance. These once established strains are generally difficult to maintain due to high mortality in early larval stages [23]. This might be by pleiotropy of the selected alleles or hitchhiking of deleterious alleles at other linked loci not specifically related to resistance.

In this study we analyzed the fitness cost of the *kdr* mutation in *Ae. aegypti*, containing both Val1016Ile and Phe1534Cys substitutions. Differently from other reports, and aiming to avoid interference of other resistance mechanisms eventually co-selected with the *kdr* mutation, we selected neither a *kdr* strain from a field population nor a laboratory strain under insecticide pressure. Instead, we assembled crosses of randomly chosen mosquitoes belonging to a field population already exhibiting a high incidence of the 1016Ile mutant allele (42,5%) [20]. The resulting CIT-32 strain was homozygous for the *kdr* allele but also presented altered GST and esterase profiles, enzymes possibly enrolled in pyrethroid metabolic resistance [31]. In this sense and in order to guarantee unbiased comparisons of life-history trait parameters between the two strains, the CIT-32 strain genetic background was enriched with that of Rockefeller (Rock), a reference susceptibility and vigor strain which has been kept under laboratory conditions for many decades [32]. This procedure originated the Rock-kdr strain. The biochemical assay revealed that major esterase and GST activities were greatly reduced in Rock-kdr, although some pNPA-esterase and MFO remained. Contrastingly, pyrethroid resistance prevailed, with a resistance profile similar to CIT-32, suggesting the *kdr* mutation is indeed the major factor contributing to resistance, although we cannot exclude possible effects associated with closely linked genes. These results indicated that the developed Rock-kdr strain, pyrethroid resistant by target site alteration, was ready to be compared to Rock in order to specifically evaluate the *kdr* mutation fitness in an environment free of insecticide. Although our aim was to develop a *kdr* homozygous mutant lineage in the 1016 site, we later realized that the 1534 position also harbored a mutation in the original couple (1016 Ile +1534 Cys) that gave rise to the CIT-32 and Rock-kdr strains, suggesting that the two mutations are in linkage disequilibrium in the wild. Therefore, all the analyses we carried out accessed the combined fitness cost of both *kdr* mutations in this double mutant allele.

Two main mechanisms, extensively studied in *Culex* mosquitoes, are commonly associated with fitness costs, resource based trade-offs and oxidative stress [26], [33], [34]. This is easy to understand in the case of metabolic resistance, since an increased production of detoxifying enzymes likely implies commitment of resources that would be important for aspects of the fitness such as longevity and fecundity. Impairment of some life history traits has been noted in organophosphate resistant *Culex* mosquitoes, such as decrease in the overwintering survival, reduced adult size, increased predation, longer developmental time and decreased male reproductive success, as reviewed elsewhere [35]. These examples are likely to derive from a trade-off between energetic resources and insecticide resistance since, for instance, an extreme over production of esterases was involved in most of the cases [12]. The affected parameters noted herein, however, are probably not related to deviation of resources originally destined to development and reproduction since we dealt with a single nucleotide polymorphism in the coding region of the sodium channel molecule, the pyrethroid target site. It cannot be ruled out that some gene variant involved with an unexplored characteristic hitchhiked together with the mutant sodium channel allele in the process of selection. However, in the lack of such evidence, it seems more parsimonious to assume that the *kdr* mutation itself was directly linked to the fitness costs here presented in the absence of pyrethroid.

In a very recent study [24], a laboratory selection for pyrethroid resistance starting from field *Ae. aegypti* populations with different resistance profiles showed consistent frequency increase of the 1016 Ile *kdr* allele. Its frequency was negatively correlated with the number of detoxifying genes differentially expressed (regardless of the down or up-regulation). The authors suggested that selection pressure for pyrethroid resistance favored the *kdr* mutation rather than metabolic alterations. Besides increase in the expression of two sigma class GST and at least 10 CYP genes related to metabolic resistance, the authors argued that there was weak selection for additional metabolic resistance genes in the insects previously “protected” with the *kdr* mutation [24].

It is not possible to generalize the resistance pleiotropic effects as negative, mainly when vector-parasite relationship is involved. For instance in *Culex quinquefasciatus* populations from Sri Lanka, a strong negative correlation was found between esterase activity accounting for organophosphate resistance and levels of the filariasis worm, *Wuchereria bancrofti* [36]. It was proposed that upregulation of carboxylesterases in response to insecticide resistance might also improve the insect immune system against pathogens [25]. Concerning *kdr* mutations, a study covering spatial and seasonal variation of malaria in Uganda showed the Leu1014Ser mutation frequency was significantly higher in *An. gambiae* infected with *Plasmodium falciparum*. The authors correlated the mutation with an increased adult longevity, resulting in higher chances of infection [37].

Target site resistance, as is the case herein, can directly influence behavioral aspects of the insect. For example, when exposed to a temperature gradient, houseflies with susceptible genotypes prefer warmer temperatures whilst individuals with the classical *kdr* mutation, Leu1014Phe, have no preferences [38]. The most striking results correlating *kdr* mutation with fitness cost were developed with the peach-potato aphids, *Myzus persicae*. *Kdr* insects presented reduced answer to the alarm pheromone, affecting the response to external stimuli as the presence of parasitoids [39]. Our *Ae. aegypti* Rock-kdr strain maintained the normal circadian activity, but the females’ locomotor activity was significantly increased. Although it is difficult to say whether this might cause an increase or decrease in fitness, as it probably depends on the specific environmental conditions, it is likely to have potential epidemiological consequences. Recently, it has been shown that infection with dengue virus also increases the locomotor activity of *Ae. aegypti* females [40]. It was assumed that this altered behavior might be translated into an increased biting rate displayed by infected mosquitoes which, based on a mathematical model, could result in dengue outbreaks with higher incidence of primary and secondary infections with severe biennial epidemics [41]. Hence, this behavioral change may directly influence traits directly related to vectorial capacity, e.g. longevity, blood feeding, intraspecific competition for resources and reproduction, here explored.

An extensive review concerning pleiotropic effects of insecticide resistance mechanisms influencing insect vector capacity, either positively or negatively, was recently presented [26]. Nevertheless, *kdr* mutations were not cited in any context. We demonstrated that the *kdr* mutations (1016 Ile +1534 Cys) have a fitness cost in *Ae. aegypti*. Among several evaluated life-history trait parameters, the Rock-kdr strain presented some negative effects on larval development timing and reproductive aspects. In the field, a delay of larval development up to the adult stage is crucial. A slower development increases the chances of larvae predation, parasitism or even breeding site destruction. Moreover, larval developmental kinetics can be related to vector density, one of the determinant aspects of the vectorial capacity [42], [43]. Under laboratory conditions, there was no significant mortality during the immature phase, and the resulting adults presented the same body weight and equivalent adult longevity when compared to the reference strain. Although the load of ingested blood did not differ between Rock and Rock-kdr females, the latter displayed reduction in the rate of insemination and number of laid eggs. This was surprising, since the number of eggs is generally directly related to the amount of ingested blood [28], [44]. Notwithstanding, Rock-kdr larvae succeeded to hatch normally from inseminated eggs. Taken together, our results suggest that the *kdr* mutation does not interfere with embryonic development itself but with fecundity.

Since rearing conditions are usually optimized in the laboratory, the fitness costs of some parameters here evaluated could be underestimated. Moreover, it is known that *Ae. aegypti* shows strong phenotypic responses to larval competition [45], [46]. For instance, when reared under high larval density, the immature development time was longer, and the adults, besides being smaller and lighter, had reduced longevity [46]. In this sense, we also evaluated some life-history trait parameters under more stringent conditions and under inter-strain competition between susceptible and resistant *kdr* genotypes. Larvae from Rock-kdr and Rock genotypes were reared together under high larval density and limited food supply conditions, in parallel with controls consisting of only one genotype under the same conditions. The Rock-kdr larvae took more time to develop when competing with Rock, compared to the controls reared alone. We suggest that, despite larval density being the same in both situations, Rock-kdr larvae are less skilled to compete with Rock for food and space and the latter end up developing faster. In other words, the susceptible genotype was more competitive in terms of development timing. In this sense, simple food resource sharing implies additional and indirect costs. Competition derived fitness costs might be related to: i) higher accumulation of nitrogenous wastes at higher densities, ii) increased physical contact which might induce stress and iii) faster resource depletion due to a higher feeding rate and a reduced energetic efficiency [46]. In the present work, whichever were the effects impairing the normal fitness, we showed that individuals homozygous for the *kdr* mutations (and possibly other closely linked loci) were more susceptible to stringent conditions than their wild-type counterparts.

Mating, copulation and finally, insemination efficiency are key factors in species whose females are inseminated only once during their lifespan, even though they have the potential to mate several times, such as *Ae. aegypti* [47]. In these cases, males must be able to compete for copula, since the first to inseminate the female will increase the chance of propagating its genes. In *Culex pipiens* mosquitoes, males from a susceptible strain exhibited an advantage over males bearing three distinct organophosphate resistant genotypes, when competing for mating with both insecticide susceptible or resistant females. Apart from specific resistance traits, all these strains shared the same genetic background, corroborating the relevant role of each evaluated resistance mechanism in this reproductive disadvantage [48]. Evolution of the *kdr* genotype and its effects on reproductive aspects have been better studied on the peach-potato aphid *Myzus persicae*, with at least one record of reproductive potential impairment of a *kdr* strain [49]. Here, we compared the ability of Rock-kdr and Rock males to inseminate females from both strains. Since Rock-kdr exhibited altered locomotor activity and reduction in the rate of inseminated females and in the number of eggs, one might expect differences in the competition for insemination. However, no differences between susceptible and resistant males were noted.

Despite no apparent cost on mating/insemination potential having been noted for the Rock-kdr strain, population cage assays definitively confirmed the fitness cost of the *kdr* mutation. In 15 consecutive non-overlapping generations, the 1016 Ile allele frequency dropped from 75 or 50 to less than 30% in most cages. The longer developmental time and the reproductive disadvantages of the Rock-kdr strain certainly contributed to this profile.

Here we presented evidences of a consistent fitness cost of the *Ae. aegypti kdr* mutations (1016 Ile +1534 Cys) in homozygosis when exposed to an insecticide free environment, although as mentioned before we cannot exclude a possible effect of closely linked loci. The assays included the dynamics under competition with a susceptible genotype, when the mutant allele tendency to decrease in frequency became evident. These results are important for insecticide management activities, mainly those based on pyrethroids. Although this is presently the preferred class of insecticides, pyrethroid use is growingly precluded due to fast resistance dissemination in *Ae. aegypti* natural populations [21]–[23]. It is expected that wild-type *Na_V_* alleles could overlap *kdr* mutations in the absence of selection pressure with pyrethroids. However, the frequency of *kdr* mutations tends to increase very rapidly under positive selection [7]. Additionally, under continued selection pressure, it is likely that the fitness cost attributed to *kdr* mutations tends to decrease due to co-selection of modifier genes [35], [50]. In this sense, enhanced surveillance for resistance should be a priority in areas where the *kdr* mutations are found. We are presently interested in determining the contribution of the isolated *kdr* alleles at positions 1016 and 1534 to both pyrethroid resistance and their related fitness cost.

## Materials and Methods

### 1. Strains Establishment

#### Isolation of 1016 Ile/Ile kdr homozygous strains

Larvae and adult mosquitoes were reared according to standard conditions previously described [51]. Mosquitoes from Cachoeiro do Itapemirim (CIT), Espírito Santo State, at the Southeastern Brazil, were chosen due to previous detection of the resistant allele at a high frequency in that locality [20]. The approach consisted of obtaining eggs from isolated couples. After genotyping all the adults, the offspring of those 1016Ile/Ile homozygous parents were selected to proceed up to the next generation. To assure virgin females would be used in the initial crosses, F1 specimens from Cachoeiro do Itapemirim, collected as described elsewhere [51], were reared until pupae, which were transferred to individual chambers. A total of 68 crosses were assembled with one male and three virgin females each inside 50 mL conical mesh covered tubes. The tubes were kept for at least three days, the insects fed *ad libitum* with a 10% sugar solution soaked cotton. One day after the sugar solution removal, females were allowed to feed on blood from anesthetized mice. After additional three days, females were individually induced to lay eggs in small Petri dishes lined with wet filter papers [52], as detailed further in the section 2.5. Meanwhile, males were genotyped for the 1011 and 1016 sites of the *AaNa_V_*, as described below. After egglaying, those females inseminated by 1016 Ile/Ile (mutant) and 1011 Ile/Ile (wild) homozygous males were also genotyped. Only eggs from crosses of the desired genotype, both parents homozygous 1011 Ile/Ile +1016 Ile/Ile, were induced to hatch. The progeny of one of the females used in cross number 32 originated strain CIT-32, which was further used in bioassays and biochemical tests. The CIT-32 strain was also adopted to establish the purified Rock-kdr strain, as stated below.

In order to isolate the 1016 Ile/Ile mutation from other potential insecticide resistance mechanisms present in the original field population, backcrosses of CIT-32 and Rockefeller (Rock), a reference strain for vigor and insecticide susceptibility [32] were performed. The heterozygous offspring of Rock and CIT-32 was allowed to copulate and lay eggs. New isolated couples were then assembled as above and only eggs derived from heterozygous parents were induced to hatch. At the ninth generation, in order to restore the homozygous genotype for the *kdr* mutation, couples were assembled with 1016 Ile/Ile specimens, originating the strain herein named Rock-kdr. The Rock-kdr strain is then homozygous for the 1016 mutation but is expected to carry a general background genotype susceptible to insecticides, similar to Rockefeller mosquitoes. The F1 offspring between Rock-kdr females and Rock males (Hib-F1), heterozygous for the 1016 site (1016 Ile/Val), was also employed in some assays.

### 2. Metabolic Resistance Assays

The activity of the main enzymes related to metabolic resistance: glutathione-S-transferases (GST), mixed function oxidases (MFO) and esterases (EST), were evaluated in one-day-old adult females. With respect to esterases, substrates α-naphthyl, β-naphthyl and p-nitrophenyl acetate (herein referred to as α-EST, β-EST and pNPA-EST) were employed. Assays for each specimen and enzyme were performed in duplicate samples, in 96 microtiter plates, totaling 35–45 Rockefeller, 41–45 CIT-32, 39–45 Hib-H1 and 58–60 Rock-kdr individuals, according to the enzyme activity evaluated. Standard susceptible profiles were taken from Rockefeller values. Mosquitoes from this reference strain were also included in all plates as internal controls. Details of reaction and analysis are extensively described elsewhere [11], [53].

### 3. Bioassays

The established strains (CIT-32, Rock-kdr and Rock) and Hib-F1 mosquitoes were submitted to dose-response bioassays in order to evaluate their profile of susceptibility to the pyrethroid, deltamethrin. The test was adapted from World Health Organization (WHO) and consists in confining mosquitoes in acrylic chamber tubes internally lined with Whatman grade n°1 papers [54] that had been previously impregnated in the lab with deltamethrin concentrations ranging from 4.2 to 1,050.0 µg/paper. Approximately 20 females, around three-days-old, were exposed to the insecticide for 1 hour and then transferred to insecticide free rescue tubes, mortality scored 24 hours later. Three replicates/dosage were used, and each assay was executed three times. Control conditions, consisting of acrylic chambers lined only with paper impregnated with the solvent (silicone oil), were run in parallel.

### 4. Evaluation of Developmental Parameters

All parameters were evaluated by simultaneously comparing Rock and Rock-kdr, reared under identical conditions, such as initial larval density and feeding, temperature and illumination regimens. Eggs were induced to hatch for approximately 24 hours. Three replicates of 500 newly emerged larvae were then randomly transferred to plastic trays (30×21×5 cm) with 1 L dechlorinated water and a 0.5 g of cat food (Friskies®, Purina, São Paulo/SP). New food supplement was offered every two days.

#### Larval development time and pupae formation

The kinetics of pupae formation under the above conditions was accompanied daily as indicative of larval development time. This assay was performed three times.

#### Adult longevity

Adults resulting from the item above, three to seven days after emergence, were randomly pooled in cylindrical cardboard cages (18×30 cm) and submitted to two alternative food regimens: 1) groups of 50 couples received sugar solution *ad libitum* as the only food source and 2) groups of 50 females, without males, received blood meals in addition to sugar solution. In this case, anesthetized mice were offered during 30 minutes on the second and 11^th^ days after cage assembling. Mortality was scored every two or three days for approximately two months. This assay was conducted twice. Comparisons of survival curves were based on the Gehan-Breslow-Wilcoxon test using GraphPad Prism version 5.00.

#### Locomotor activity and circadian rhythm

These parameters were evaluated with a Locomotor Activity Monitor (TriKinetics) as described in previous studies [28], [55]. Four to five-day old females were individually placed in glass tubes with a cotton plug soaked in 10% sucrose solution and the tubes were placed in the Monitor inside a Precision Scientific Incubator Mod. 818 under constant temperature (25°C) and a 12 h light, 12 h dark photoperiod (LD 12∶12). The locomotor activity was individually registered every time a mosquito crossed the middle of the tube, interrupting an incident infrared light. For every mosquito, 48 data points (representing the total locomotor activity of 30 min intervals) were obtained for every day of monitoring. Mosquitoes were allowed to acclimatize to the conditions inside the monitor tubes for two days and data from the third up to the sixth day of locomotor activity monitoring were used in the statistical analysis. Only data from mosquitoes that were alive up to the seventh day of monitoring were considered for the analysis, which was performed through calculation of the Williams average of their activity. In each assay a total of 32 individual females of each strain were evaluated. This assay was carried out twice. For statistical analysis, pyrethroid resistant and control *Ae. aegypti* groups were compared by t test with the SPSS software.

#### Blood feeding

The amount of blood ingested by females was inferred as the weight ratio after and before the blood meal, as performed elsewhere [56]. To accomplish this, 100 three-day old females were deprived of sugar solution during 24 hours before the assay. Six to seven pools of five females each were killed (by rapid freezing) and weighed in an analytical balance (APX –200, Denver Instrument). In parallel, anesthetized mice were offered during 30 minutes to the remaining alive females. Additional six to seven groups of five engorged females were killed and weighed as above. The relative amount of ingested blood was obtained by comparing the average value of both groups. This assay was performed twice.

### 5. Reproductive Parameters

#### Female fecundity

Roughly equivalent numbers of males and females were confined in cages for at least three days before a blood meal was offered to females, as detailed above. According to our rearing conditions, this period is sufficient for insemination of all healthy females [28]. Three days after blood feeding, oviposition was induced [52]. Briefly, around 30 females from each strain were individualized in small Petri dishes (6 cm in diameter) lined with filter paper in the lids. After moistening the filter paper with 700 µL dechlorinated water, the Petri dishes remained in a humid chamber inside the insectary for two days, when the number of egglaying females and the amount of eggs/female were recorded. Virgin females generally lay a smaller number of eggs or do not lay eggs at all [28]. Females were then classified in three groups: i) those that lay no eggs, ii) females laying less than 50 eggs and iii) females that oviposit 50 or more eggs. This assay was performed twice.

#### Egg viability

Three days after blood feeding, around 100 females were transferred to a new cage, containing a black cup internally lined with filter paper and filed with dechlorinated water to receive ovipositing eggs. For each strain, three to four groups of 100 eggs, 24–48 hours old, were randomly gathered with a smooth and humid brush. Seven days after the eggs had dried, each replicate was individually submerged in 300 mL of dechlorinated water with 0.25 g of cat food (Friskies®, Purina, São Paulo/SP) during 24h, and hatching larvae were counted. This assay was performed three times.

### 6. Competition Analysis

#### Larval development time

Competition between Rock and Rock-kdr larvae was evaluated under space and food stringent conditions. Triplicates of 250 one-day old larvae of each strain were placed together in a small tray (30×18×5 cm) containing 800 mL dechlorinated water. Two pellets of cat food were added every four days. Controls consisted of trays with 500 Rock or Rock-kdr larvae under the same conditions and also in triplicate. Pupae were counted daily and transferred to cages. A total of 30% of emerged males, daily discriminated, were genotyped for the 1016 site (see [21]) in order to determine their strain.

#### Competition for mating

Virgin adults reared under laboratory standard conditions were grouped in two cages, both containing 15 Rock and 15 Rock-kdr males. Cage A was completed with 30 Rock females and cage B, with 30 Rock-kdr females. After three days, females were blood fed and individually induced to oviposit. Larvae were stimulated to hatch as aforementioned. *Aedes aegypti* females are monogamous, meaning that they are inseminated only once [29]. Nevertheless, we analyzed the genotype of several larvae of an offspring, by pooling them in three groups of seven F_1_ L3 larvae of each female, by allelic-specific PCR [21].

#### Population cage experiments

The dynamics of *kdr* frequency in the absence of insecticide pressure was achieved by cage trial assays. Each cage had an initial 1016 Ile (the mutant allele) frequency of 50 or 75%. For the first case, cages were mounted with 30 Rock-kdr females and 30 Rock males and the other with 30 Rock-kdr females and 15 Rock-kdr +15 Rock males, rendering the initial allele proportion required. Three cages with each start frequency were rigorously kept under the same lab standard conditions during 15 generations without any gene flow among them. At each generation, females fed on blood twice, the first blood meal being offered at least seven days after the end of pupation. The first oviposition was used to rear the next generation, and the second one was kept as a backup. Eggs were induced to hatch in 24 hours, and two days later, 500 larvae were randomly transferred to a new tray. Nearly 30 males in each cage were genotyped at each generation.

### 7. Statistical Analysis

The hypothesis tests for comparing the parameters between Rock and Rock-kdr were indicated in each assay methods and together with the results. Otherwise stated, graphs and analysis were performed with the software GraphPad Prism version 5.04 for Windows, GraphPad Software, La Jolla California USA.

### 8. Ethics Statement

#### Mosquito blood feeding

*Ae. aegypti* females were fed on anesthetized mice (Ketamine:Xylazine 80–120 mg/kg:10–16 mg/kg), accordingly to the institutional proceedings, [57] which is oriented by the national guideline “The Brazilian legal framework on the scientific use of animals” [58]. This study was reviewed and approved by the Fiocruz institutional committee “Comissão de Ética no Estudo de Animais” (CEUA/FIOCRUZ), license number: L-011/09.

#### Entomological survey

The egg collections at Cachoeiro do Itapemirim were conducted by agents from the Health Secretariat of Espírito Santo State, following procedures designed by the National Program of Dengue Control/Ministry of Health-Brazil. Ovitraps were installed and collected in the dwellings with the residents’ permission.

## Supporting Information

Figure S1 **Adult longevity of Rock-kdr and Rock** ***Ae. aegypti*** **strains.** Survival curves of males (A) and females (B, C) fed exclusively with sugar solution (A, B) or with sugar and two blood meals (offered at days 2 and 11).
(TIF)

Figure S2 **Blood feeding.** Each dot represents a pool of females weight before and after blood feeding. Median and SE were evidenced.
(TIF)

Table S1 Competition analysis, development time until adult. Number of daily emerged males.
(PDF)

Table S2 Competition analysis, development time until adult. Number of individuals collected from each tray and observed genotypes.
(PDF)

Table S3 Population cage experiments. Numbers of individuals genotyped from each cage throughout generations.
(PDF)
