# Supplementary figures and images for: Assessing the Effects of Aedes aegypti kdr Mutations on Pyrethroid Resistance and Its Fitness Cost
Source: PLoS One. 2013 Apr 8;8(4):e60878. doi: 10.1371/journal.pone.0060878 (PMC3620451; doi:10.1371/journal.pone.0060878)

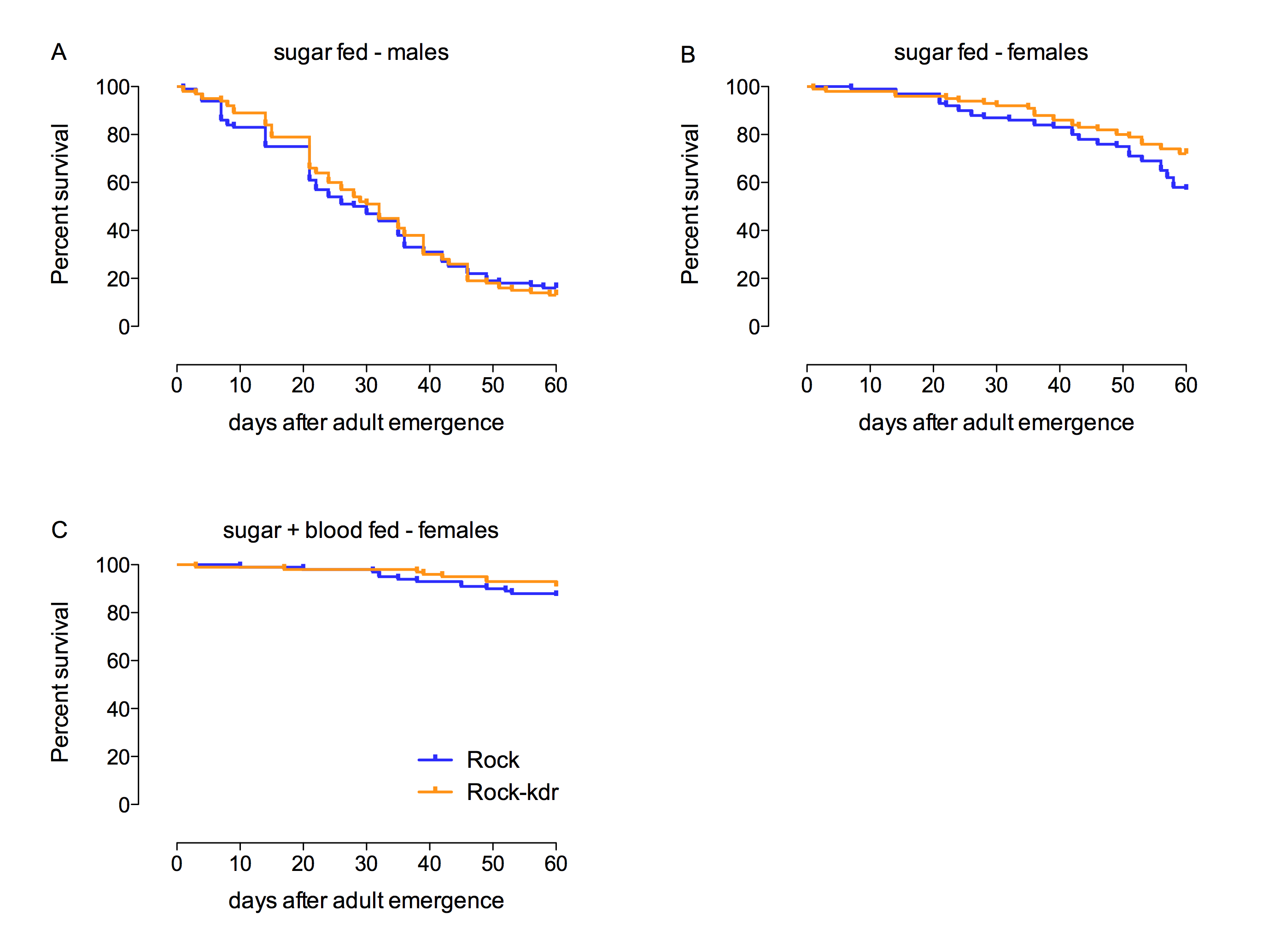

Supplement: Figure S1 — Adult longevity of Rock-kdr and Rock Ae. aegypti strains. Survival curves of males (A) and females (B, C) fed exclusively with sugar solution (A, B) or with sugar and two blood meals (offered at days 2 and 11). (TIF) [file pone.0060878.s001.tif]

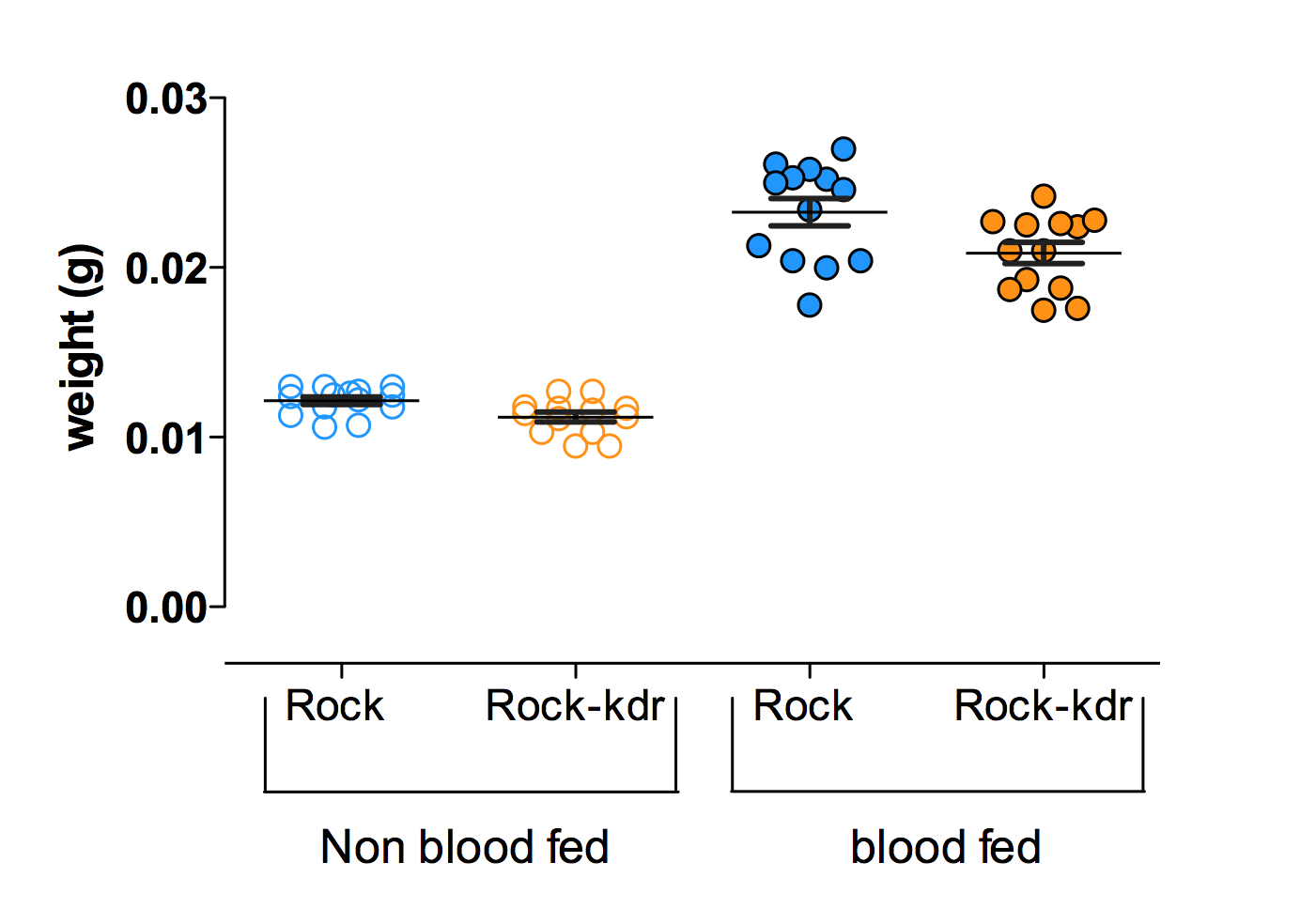

Supplement: Figure S2 — Blood feeding. Each dot represents a pool of females weight before and after blood feeding. Median and SE were evidenced. (TIF) [file pone.0060878.s002.tif]
