# Supplementary material for: Assessing the Effects of Aedes aegypti kdr Mutations on Pyrethroid Resistance and Its Fitness Cost
Source: PLoS One. 2013 Apr 8;8(4):e60878. doi: 10.1371/journal.pone.0060878 (PMC3620451; doi:10.1371/journal.pone.0060878)
Supplement: Table S1 — Competition analysis, development time until adult. Number of daily emerged males. (PDF) [file pone.0060878.s003.pdf]

Table S1. Competition analysis, development time until adult. Number of daily emerged males.

	Daily emerged males (pooled trays)		
day	Rock	Aa-kdr	Rock + Aa-kdr
1	25	23	67
2	162	51	159
3	120	179	146
4	174	117	110
5	83	103	115
6	40	40	35
7	21	56	37
8	7	19	18
