# Supplementary material for: Assessing the Effects of Aedes aegypti kdr Mutations on Pyrethroid Resistance and Its Fitness Cost
Source: PLoS One. 2013 Apr 8;8(4):e60878. doi: 10.1371/journal.pone.0060878 (PMC3620451; doi:10.1371/journal.pone.0060878)
Supplement: Table S2 — Competition analysis, development time until adult. Number of individuals collected from each tray and observed genotypes. (PDF) [file pone.0060878.s004.pdf]

Table S2. Competition analysis, development time until adult. Number of individuals collected from each tray and observed genotypes.

day	30% males/ tray			n genotyped	
	tray 1	tray 2	tray 3	Val/Val	Ile/Ile
1	4	8	8	20	0
2	16	26	7	37	12
3	16	14	14	17	26
4	11	14	8	12	20
5	10	7	18	7	17
6	4	2	4	3	3
7	3	5	3	1	10
8	3	2	0	1	4

Developmental competition between larvae from Rock and Aa-kdr Ae. aegypti lineages’
