# Supplementary material for: Assessing the Effects of Aedes aegypti kdr Mutations on Pyrethroid Resistance and Its Fitness Cost
Source: PLoS One. 2013 Apr 8;8(4):e60878. doi: 10.1371/journal.pone.0060878 (PMC3620451; doi:10.1371/journal.pone.0060878)
Supplement: Table S3 — Population cage experiments. Numbers of individuals genotyped from each cage throughout generations. (PDF) [file pone.0060878.s005.pdf]

Table S3. Population cage experiments. Numbers of individuals genotyped from each cage throughout generations.

Cages	Genotype	Number of individuals/ genotype on generations				
		3rd	6th	9th	12th	15th
1	Val/Val	12	14	20	19	16
	Val/Ile	11	11	5	8	9
	Ile/Ile	6	5	2	3	5
2	Val/Val	13	10	12	13	23
	Val/Ile	11	14	5	7	6
	Ile/Ile	6	6	13	5	1
3	Val/Val	13	10	12	13	23
	Val/Ile	11	14	5	7	6
	Ile/Ile	6	6	13	5	1
4	Val/Val	4	9	19	17	14
	Val/Ile	13	12	7	9	14
	Ile/Ile	13	8	4	4	2
5	Val/Val	8	3	5	9	20
	Val/Ile	13	7	6	10	3
	Ile/Ile	6	5	11	11	6
6	Val/Val	11	10	16	9	28
	Val/Ile	17	13	9	10	2
	Ile/Ile	2	7	5	11	0
